# Impact of Increased End-Tidal Carbon Dioxide on Continuous Noninvasive Hemoglobin Monitoring during Laparoscopic Gastrectomy: A Randomized Controlled Study

**DOI:** 10.3390/jpm12020160

**Published:** 2022-01-26

**Authors:** Ha-Yeon Kim, Jong-Bum Choi, Sang-Uk Han, Hye-Sun Lee, Kyuhyeok Lee, Ji-Eun Kim

**Affiliations:** 1 Department of Anesthesiology and Pain Medicine, Ajou University School of Medicine, Suwon 16499, Korea; hayeon@aumc.ac.kr (H.-Y.K.); romeojb@aumc.ac.kr (J.-B.C.); mybfgg@gmail.com (K.L.); 2 Department of Surgery, Ajou University School of Medicine, Suwon 16499, Korea; hansu@aumc.ac.kr; 3 Biostatistics Collaboration Unit, Yonsei University College of Medicine, Seoul 03722, Korea; HSLEE1@yuhs.ac

**Keywords:** hemoglobins, hypercapnia, laparoscopy, oximetry, transcutaneous blood gas monitoring

## Abstract

The pulse CO-Oximetry allows continuous, noninvasive monitoring of hemoglobin (SpHb). We assessed the impact of increased end-tidal carbon dioxide (EtCO_2_) on the accuracy and trending ability of SpHb in laparoscopic surgery. Participants (*n* = 64) were randomly allocated to the low carbon dioxide (CO_2_) group (EtCO_2_: 30–35 mmHg) or the high CO_2_ group (EtCO_2_: 40–45 mmHg). The SpHb and laboratory hemoglobin (tHb) were obtained during surgery. The correlation coefficient (r) between SpHb and tHb showed greater tendency in the low CO_2_ group (r = 0.68) than in the high CO_2_ group (r = 0.43). The bias (precision) was −1.18 (1.09) with a limit of agreement (LOA) of −3.31 to 0.95 in low CO_2_ group and −1.02 (1.24) with a LOA of −3.45 to 1.42 in high CO_2_ group; they did not differ significantly between the groups (*p* = 0.246). The low CO_2_ group showed a high concordance rate of 95.9% and a moderate correlation between ΔSpHb and ΔtHb (r = 0.53). However, the high CO_2_ group showed a concordance rate of 77.8% and no correlation between ΔSpHb and ΔtHb (r = 0.11). In conclusion, increased EtCO_2_ significantly reduced the trending ability of SpHb during laparoscopic surgery. Caution should be executed when interpreting SpHb values during laparoscopic surgery in patients with hypercapnia.

## 1. Introduction

To date, laparoscopic surgery has expanded to various surgeries because of its minimal invasiveness, few complications, and rapid recovery. Despite numerous benefits, pneumoperitoneum is required during laparoscopic surgery to secure the surgical view; however, it is associated with some disadvantages, such as excessive carbon dioxide (CO_2_) retention and underestimated blood loss because of the limited view through the scope [1]. Moreover, pneumoperitoneum stimulates the sympathetic nervous system, leading to hemodynamic changes [2], which may be confused with changes of vital signs related to blood loss. Thus, rapid assessment of the hemoglobin (Hb) concentration is essential even in laparoscopy.

The Radical-7 pulse CO-Oximetry is a device that allows monitoring of continuous, noninvasive hemoglobin (SpHb). It was developed in the process of overcoming pulse oximetry’s shortcomings and uses 8-wavelength spectrometry, whereas pulse oximetry uses 2-wavelength spectrometry [3]. Compared to a laboratory Hb (tHb), SpHb enables real-time monitoring of Hb changes without invasiveness, time-delay, unnecessary labor, and collection of serial blood samples. Considering that one-third of transfusions in the operation room are administered without first obtaining tHb value [4], SpHb can help to minimize unnecessary blood sampling and transfusions along with reducing the associated costs and improving the quality of care, which is a major public health issue [5,6].

Several clinical studies have investigated the accuracy, precision, and trending ability of SpHb in various conditions including indigo carmine, anemia, cardiopulmonary bypass, transplantation, trauma, dark-skinned and hemodilution [7,8,9,10,11,12]. In a previous study, altered arterial CO_2_ pressure (PaCO_2_) impaired the agreement between oxygen saturation calculated from arterial blood analyzers and that measured with pulse oximetry [13]. Pulse oximetry and pulse CO-Oximetry have technological similarity based on light absorption through a finger sensor. Recently, an observational pilot study suggested that the values of SpHb may be affected by the presence of CO_2_ insufflation [14]. Therefore, we hypothesized that increased end-tidal CO_2_ (EtCO_2_) would affect sensing of SpHb and reduce the accuracy and trending ability of SpHb during laparoscopic surgery. This study aimed to assess the impact of increased EtCO_2_ on the accuracy and trending ability of SpHb in patients undergoing laparoscopic gastrectomy.

## 2. Materials and Methods

### 2.1. Study Design and Patients

This randomized, double-blinded, controlled study was conducted at Ajou University Health System. The present study was approved by the Ajou Hospital Institutional Review Board (AJIRB-MED-OBS-17-339, 8 January 2018) and registered prior to patient enrollment at ClinicalTrial.gov (NCT03430778, principal investigator: JEK, registration date: 13 February 2018). Written informed consent was obtained from all participants. This manuscript adheres to the applicable CONSORT guidelines.

Patients undergoing elective laparoscopic gastrectomy with an American Society of Anesthesiologists physical status I-III, aged 19–85 years, were included. Exclusion criteria were prior surgery involving the hand, infection, hematologic diseases, or refusal to participate in this study.

### 2.2. Interventions

Participants were randomly allocated to one of the two groups by computer-generated randomization (http://www.random.org, 14 February 2018): the low CO_2_ group or the high CO_2_ group. The low CO_2_ group participants were maintained at an EtCO_2_ level of 30–35 mmHg by adjusting the tidal volume and inspiratory rate within a peak inspiratory pressure ≤ 29 cmH_2_O during surgery. The high CO_2_ group participants were maintained at an EtCO_2_ level of 40–45 mmHg during surgery. Group assignment information was concealed in a sealed, opaque envelope. Before anesthesia induction, the envelope was opened by an independent investigator who performed all interventions but was not involved in outcome assessment (J.B.C.). He adjusted the respiratory rate according to the group assignment in all patients. The outcome assessor who did not perceive the concept of study conducted the blood sampling and data recording (K.L.). The other investigators who did not know the patient’s group assignment collected the data of the blood analyzer from electrical medical records (H.Y.K. and J.E.K).

On patients’ arrival to the operating room, basic monitoring (pulse oximetry, electrocardiography, and non-invasive blood pressure measurement) was performed. A spectrophotometric adhesive SpHb sensor (Rainbow R1 25, Rev E; Masimo, Irvine, CA, USA) was applied to the patients’ fourth finger and was covered with an impermeable black shield. SpHb levels were continuously monitored with a Radical-7 Pulse CO-Oximetry (Masimo, Irvine, CA, USA; software version 1451). Anesthesia was induced with intravenous (IV) propofol (2 mg/kg) and remifentanil (3.0–4.0 ng/mL) using a target-controlled infusion, followed by rocuronium (1 mg/kg). After endotracheal intubation, mechanical ventilation was initiated with a tidal volume of 8 mL/kg and an inspired oxygen fraction (FiO_2_) of 0.5. Anesthesia was maintained with a remifentanil (target concentration, 0.5–4.0 ng/mL), sevoflurane (2–2.5%), and rocuronium infusion within a surgical pleth index of 30–50, bispectral index score of 40–60, and train-of-four of 1–2. Lactate Ringer’s solution was infused at a rate of 5–10 mL/kg/h. A radial arterial catheter was placed on the contralateral side of patients as the SpHb sensor. The arterial blood was sampled to measure tHb using the satellite CO-Oximetry (Stat Profile pHOx Ultra; Nova Biomedical, Waltham, MA, USA). In case of the mean arterial pressure (MAP) < 60 mmHg, an IV bolus of ephedrine (4 mg) was primarily administered, and infusion of norepinephrine was administered as needed. At the end of the surgery, an IV of propacetamol (1 g) and ramosetron (0.3 mg) was administered for postoperative analgesia and antiemetic treatment. After confirming the train-of-four ≥ 2 using a nerve stimulator, an IV of sugammadex (2 mg/kg) was administered for reversal of the neuromuscular block. After confirming an adequate tidal volume, patients were extubated and transferred to a post-anesthesia care unit.

### 2.3. Data Collection

Arterial blood sampling was obtained after confirming that SpHb was unchanged for 30 s. Simultaneous SpHb and the perfusion index (PI) were recorded within 10 s after arterial blood sampling. In vivo adjustment (calibration) was not conducted. Hemodynamics (heart rate [HR] and MAP), data on the monitor (SpHb, PI, and EtCO_2_), and data from the blood analyzer (pH, PaCO_2_, arterial oxygen pressure [PaO_2_], tHb, and bicarbonate) were collected at five time points: after anesthesia induction; at 30, 60, and 90 min after pneumoperitoneum; and at the end of the surgery. Intraoperative vasopressor use was also recorded.

### 2.4. Statistical Analysis

The primary end point was the bias (=SpHb–tHb). The sample size was calculated based on the bias. In a previous study, the standard deviation (SD) of bias during use of pulse CO-Oximetry was 0.92 g/dL [15]. Considering that a mean difference of 0.8 in bias was significant, 28 participants were required in each group for a significance level of 5% and a power of 90%. Considering the dropout rate of 25%, 70 patients were included.

All paired data (SpHb–tHb) and changes in SpHb (ΔSpHb) and tHb (ΔtHb) between two consecutive measurements were analyzed. SpHb values were excluded in the analysis when the PI was <1, because a low PI affects the accuracy.

Correlation between simultaneous SpHb and tHb measurement pairs was depicted in a scatter plot, and coefficients of determination (r values) were calculated by Pearson correlation analysis. For the accuracy of SpHb, a modified Bland—Altman’s method was used with consideration of multiple measurements per individual. The precision was defined as 1 SD of the bias. The 95% limits of agreement (LOA) were presented by calculating the interval defined by the bias ± 1.96 SD. The correlation coefficient between SpHb and tHb with repeated observations for each patient was estimated by using a mixed-effects model. For trend analysis, the four-quadrant plot was used with differences of ΔSpHb and ΔtHb. A central exclusion zone of 1 g/dL was applied to compensate for intrinsic SpHb bias. The effect of PaCO_2_ on the bias and SD was analyzed using the F test and *t* test. The relationship between bias and PaCO_2_ was analyzed with a mixed-effects model for different PaCO_2_ ranges (PaCO_2_ < 35 mmHg, 35 mmHg ≤ PaCO_2_ < 40 mmHg, and PaCO_2_ ≥ 40 mmHg).

Data are presented as mean ± SD [range], median [range], or number of patients (%). Normality of distribution was assessed with the Shapiro–Wilk test. Parametric and nonparametric data were analyzed using the Student’s t-test and Mann–Whitney test. Categorical data were compared using the chi-square test or Fisher’s exact test. Repeated measured data were analyzed using the linear mixed model and post-hoc analysis. When the interaction was statistically significant, the *p*-value was adjusted with Bonferroni correction. A *p* value < 0.05 was considered statistically significant. Statistical analysis was conducted with SPSS for Windows (version 25.0; IBM Corp., Armonk, NY, USA) and SAS (version 9.4; SAS Inc., Cary, NC, USA).

## 3. Results

### 3.1. Study Population and Intraoperative Characteristics

**Figure 1:**
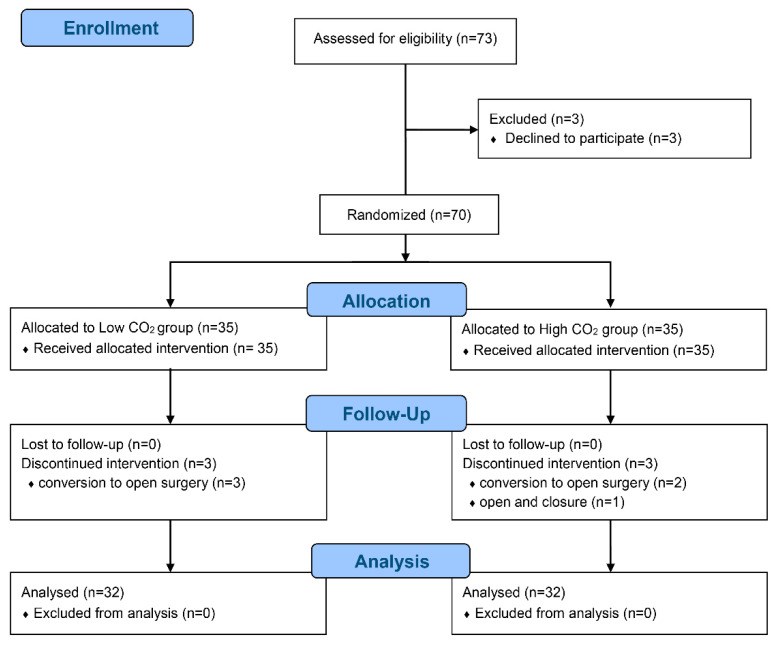
CONSORT flow diagram.

Seventy patients were enrolled and randomized between January 2019 and May 2019, but six patients withdrawn because of conversion to open surgery (*n* = 5) and closure due to abdominal seeding (*n* = 1) (Figure 1).

There were no significant differences in patient characteristics and surgical data between the high and low CO_2_ groups (Table 1). No patient received a transfusion during surgery. Both HR and MAP were adequately maintained and comparable between the groups (P_group×time_ = 0.787 and P_group×time_ = 0.423, respectively). The PaO_2_ and pH were also comparable between the groups (P_group×time_ = 0.423 and P_group×time_ = 0.138); however, PaCO_2_ and bicarbonate were significantly higher in the high CO_2_ group than in the low CO_2_ group at all time points (P_group×time_ < 0.001 and P_group×time_ = 0.020, respectively).

### 3.2. Collection of SpHb and tHb Values

In total, 320 paired measurements of SpHb and tHb were collected from 64 patients. Twenty-five SpHb measurements (7.8%, 13 in the low CO_2_ group and 12 in the high CO_2_ group) were excluded because the PI was <1.0. The mean PI values for the remaining SpHb measurements were 3.9 ± 2.0 [1–8.2]% in the low CO_2_ group and 4.2 ± 2.5 [1–12]% in the high CO_2_ group (Table 1). Finally, 295 paired measurements of SpHb and tHb were analyzed (92.2%, 147 in the low CO_2_ group and 148 in the high CO_2_ group). The SpHb and tHb values were significantly lower in the low CO_2_ group than in the high CO_2_ group (*p* < 0.001 and *p* = 0.003, respectively, Table 1).

### 3.3. Accuracy and Trending Ability of SpHb

**Figure 2:**
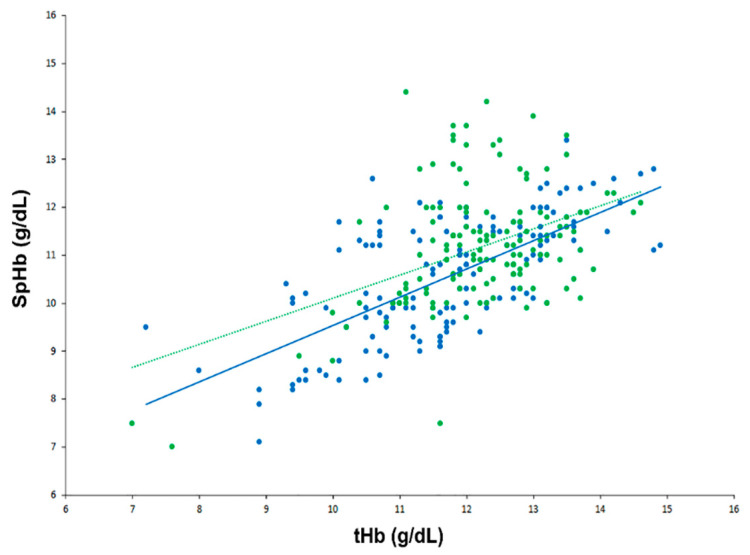
Scatter plot showing real-time continuous hemoglobin and laboratory hemoglobin data in the low carbon dioxide (CO_2_) (blue) and high CO_2_ groups (green).

**Figure 3:**
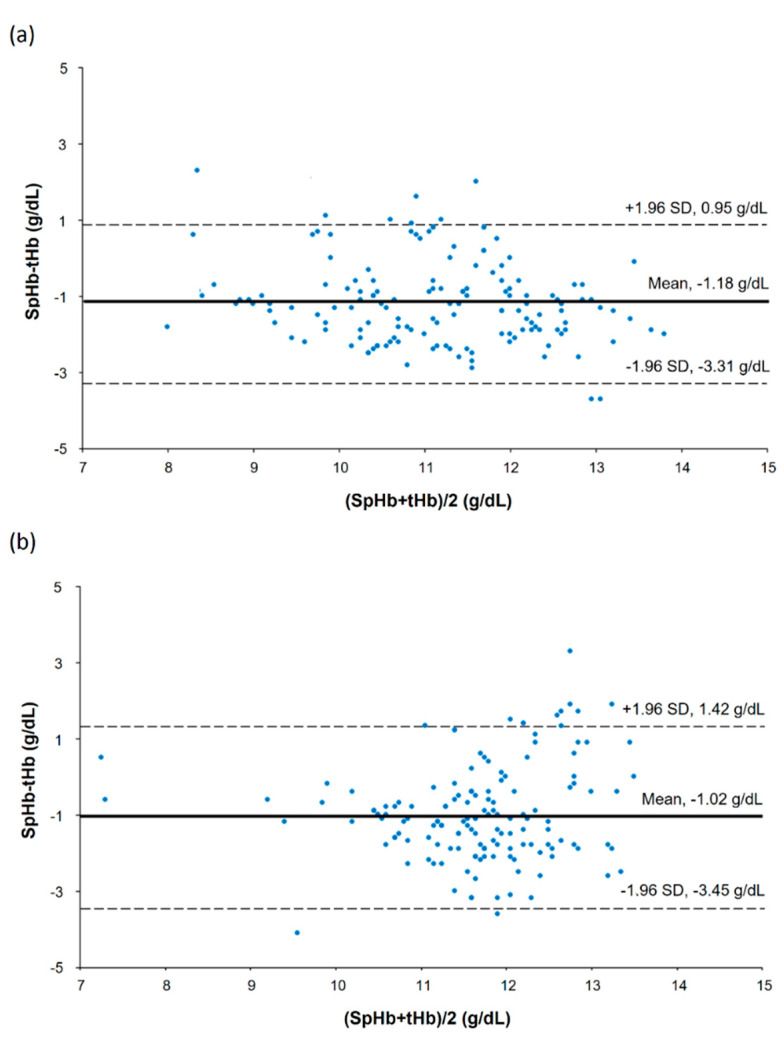
Bland—Altman’s plot showing repeated measurements in the low carbon dioxide (CO_2_) (**a**) and high CO_2_ groups (**b**).

In a scatter plot with all SpHb and tHb data points, there was a strong positive correlation between SpHb and tHb in the low CO_2_ group but a moderate positive correlation in the high CO_2_ group (Figure 2): the r values were 0.68 (95% confidence interval [CI] 0.58 to 0.76) and 0.43 (95% CI 0.29 to 0.55) for the low and high CO_2_ groups, respectively (*p* = 0.153). Results of the modified Bland–Altman’s analysis for repeated measurements are shown in Figure 3. The bias (precision) values were −1.18 (1.09) with an LOA of −3.31 to 0.95 in the low CO_2_ group and −1.02 (1.24) with an LOA of −3.45 to 1.42 in the high CO_2_ group, and they did not differ significantly between the groups (*p* = 0.246).

**Figure 4:**
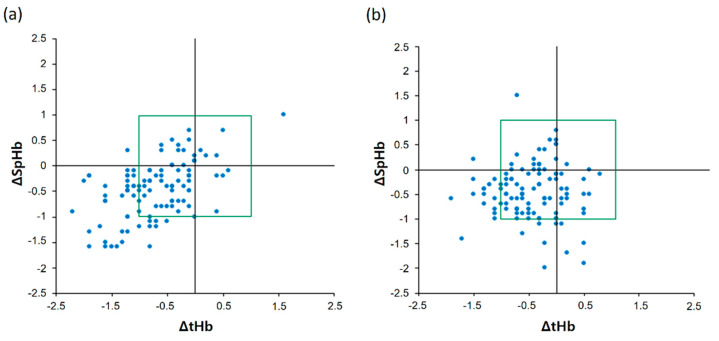
Four-quadrant plot showing changes in real-time continuous hemoglobin values and laboratory hemoglobin values in the low carbon dioxide (CO_2_) (**a**) and high CO_2_ groups (**b**).

The four-quadrant plot of ΔSpHb and ΔtHb is shown in Figure 4. The low CO_2_ group showed a highly acceptable concordance rate of 95.9% (47 of 49) and a moderate positive correlation between ΔSpHb and ΔtHb (Figure 4a): the r value was 0.53 (95% CI 0.38 to 0.65). However, the high CO_2_ group showed a lower acceptable concordance rate of 77.8% (28 of 36) than the low CO_2_ group and no correlation between ΔSpHb and ΔtHb (Figure 4b): the r value was 0.11 (95% CI −0.08 to 0.29).

### 3.4. Comparisons According to the PaCO_2_ Ranges

According to the PaCO_2_ ranges, bias was significantly increased in the PaCO_2_ ≥ 45 mmHg range compared with those in the PaCO_2_ < 35 mmHg and 35 ≤ PaCO_2_ < 45 mmHg ranges (*p* = 0.020 and *p* = 0.004, respectively; Table 2).

## 4. Discussion

To our knowledge, this randomized controlled study is the first to assess the impact of increased EtCO_2_ on SpHb during laparoscopic surgery. The bias did not differ between the groups. However, the correlation between SpHb and tHb was strong in the low CO_2_ group but moderate in the high CO_2_ group. In addition, the concordance rate between SpHb and tHb was more acceptable in the low CO_2_ group than in the high CO_2_ group. Moreover, the correlation between ∆SpHb and ∆tHb was moderate in the low CO_2_ group but negligible in the high CO_2_ group. Lastly, the bias was significantly increased in the range of PaCO_2_ ≥ 45 mmHg.

Pulse oximetry and pulse CO-Oximetry are based on the measurement of the differential optical density of light that pass through the tissue. The accuracy of pulse oximetry, when being evaluated by the difference between the oxygen saturation by pulse oximetry and that in the arterial blood, was affected by several factors [16,17]. Similarly, the accuracy of pulse CO-Oximetry has been evaluated using the SpHb and tHb values. In there, SpHb has shown clinical usefulness but was affected by various factors [7,8,9,10,11,12]. In previous studies, altered PaCO_2_ levels affected the accuracy and reliability of oxygen saturation in pulse oximetry [13]. Hence, we differentiated the EtCO_2_ levels between the groups and assessed the SpHb values.

In our study, increased EtCO_2_ did not affect the accuracy of SpHb in terms of the similar bias between the groups, although the low CO_2_ group showed a lower precision and a narrower LOA than the high CO_2_ group. The bias values in the low and high CO_2_ groups seem to be adequate in terms of accuracy, by being within ≤1.5 g/dL of the tHb as proposed by Miller et al. [18], but they were slightly high compared to a meta-analysis result of −0.27 g/dL performed in the operating room [19]. This may be because we did not conduct the calibration (in-vivo adjustment). If SpHb was calibrated with the initial tHb, the bias in our study would be deceased, because a previous study reported that after calibration, bias and the SD of SpHb were reduced by 0.5 g/dL compared to tHb in surgical patients [20].

In contrast to accuracy, increased EtCO_2_ reduced the trending ability of SpHb in our study, in which the levels of concordance and correlation between SpHb and tHb were strong enough to reduce the number of invasive blood samplings by pulse CO-Oximetry in the low CO_2_ group but less in the high CO_2_ group. Since inception of pulse CO-Oximetry in 2008, numerous studies investigating its ability have focused on the accuracy of SpHb as the absolute difference of Hb compared to conventional methods [21,22]. These studies showed reasonable accuracy for SpHb, but certain patient conditions limited this. For example, Riess et al. investigated the use of SpHb during cardiac surgery and found SpHb to be accurate before but not after cardiopulmonary bypass. They concluded that sole reliance on SpHb for deciding transfusion is inappropriate [8]. A few SpHb studies have evaluated the trending ability and accuracy of SpHb [9,10,12]. Barker et al. suggested that the primary value of SpHb is derived from a continuous, real-time measurement and trending rather than accuracy, and that SpHb is not an alternative to tHb but an additional trend monitor [23]. Downward, stable, or upward SpHb trends enable clinicians to quickly decide whether to transfuse a patient. Thus, altered trending ability of SpHb leads to time-delayed or unnecessary red blood cell transfusion. In our study, it may be clinically more meaningful that increased EtCO_2_ during laparoscopic surgery reduced the ability of trending in SpHb rather than not affecting the accuracy of SpHb.

Several possibilities may explain the reduced ability of trending in SpHb in our study. First, pulse CO-Oximetry measures the concentrations of four Hb types: oxyhemoglobin, deoxyhemoglobin, methemoglobin, and carboxyhemoglobin. Thus, increased PaCO_2_ may alter the wavelength reading because of increases in the amount of carbaminohemoglobin, considering that about 10% of the CO_2_ is carried as carbaminohemoglobin [24]. Second, PaCO_2_-related vasodilation causes dynamic circulation (opening) in an arteriovenous shunt at the fingertip [25,26], which may lead to venous pulsation and spurious detection by pulse CO-Oximetry of venous blood as arterial. It is notable because deoxyhemoglobin can carry more CO_2_ than the oxygenated form (the Haldane effect) [24]. Third, increased PaCO_2_ causes a reduction in CO_2_-medicated extracellular pH [25], which can induce changes in red blood cell morphology [27].

There are several limitations to our study. First, tHb was measured using satellite CO-Oximetry, which has been the common method to measure tHb in the operating room. However, the International Committee for Standardization in Hematology recommends a central laboratory hematology analyzer that uses the cyanide method for tHb measurement [28]. Second, the study groups were divided according to EtCO_2_, and not PaCO_2_. Although EtCO_2_ measurement is a good non-invasive method to estimate PaCO_2_, EtCO_2_ may not predict the actual PaCO_2_ in certain circumstances. Third, we used an older version of SpHb sensors (Rev E), but updated SpHb sensors may show the different results. Fourth, all patients were transferred to the operating room for a scheduled surgery with stable hemodynamics and a Hb level within the normal range. Further studies are needed considering special conditions, such as low Hb levels or critical illness. Fifth, all adhesive SpHb sensors were applied to the fourth finger of each patient’s left hand. Since differences in oxygen saturation among fingers or between dominant and non-dominant hands have been reported, this may have affected our results, although arterial catheterization was performed in the right arm and SpHb values with a PI < 1.0 were excluded [29]. Sixth, we did not calibrate the SpHb with initial tHb. However, for the assessment of ΔSpHb and ΔtHb, calibration could have been beneficial to assess a drift over the increased EtCO_2_ or the time.

## 5. Conclusions

An increased EtCO_2_ significantly reduced the trending ability of SpHb in patients undergoing laparoscopic gastrectomy. Caution should be executed during decision-making regarding transfusion based on the SpHb value in patients with hypercapnia.

## Figures and Tables

**Table 1:** Patient characteristics and perioperative profiles.

	Low CO_2_ Group (*n* = 32)	High CO_2_ Group (*n* = 32)	*p*-Value
Age (yr)	62 ± 14	61 ± 12	0.871
Height (cm)	165.6 ± 8.8	166.1 ± 7.4	0.808
Weight (kg)	67.6 ± 12.3	66.8 ± 10.6	0.800
Sex (male/female)	27/5	27/5	>0.999
ASA physical status (1/2/3)	15/14/3	15/14/3	>0.999
Diabetes	4 (13%)	8 (25%)	0.200
COPD	0	2 (6%)	0.492
Crystalloid + colloid (mL)	1575 (1453−1775)	1600 (1200−2075)	0.962
Urine (mL)	165 (110−308)	138 (95−290)	0.629
Bleeding (mL)	100 (100−150)	100 (100−150)	0.482
Infusion of norepinephrine	4 (13%)	6 (19%)	0.491
Infusion of isosorbide dinitrate	2 (6%)	2 (6%)	>0.999
Perfusion index (%)	3.9 ± 2.0	4.2 ± 2.5	0.145
SpHb (g/dL)	10.6 ± 1.2	11.2 ± 1.2	<0.001
tHb (g/dL)	11.7 ± 1.4	12.2 ± 1.1	0.003
EtCO_2_ (mmHg)	31.8 ± 2.3	42.0 ± 2.9	<0.001
PaCO_2_ (mmHg)	32.0 ± 3.2	42.3 ± 4.6	<0.001
Pneumoperitoneum time (min)	131 (116−145)	119 (98−156)	0.383
Anesthesia time (min)	198 ± 37	188 ± 46	0.351
PACU profiles			
Pain score	7 (6−8)	7 (6.5−9)	
Nausea (1/2/3/4)	2/26/3/0	1/30/1/0	
Vomiting	0	1 (3%)	
Antiemetics	3 (10%)	1 (3%)	
Analgesics	29 (94%)	30 (94%)	
Duration of PACU stay (min)	50 (40−60)	50 (40−55)	

Values are presented as mean ± standard deviation, median (interquartile range) or number of patients (%). ASA, American Society of Anesthesiologists; COPD, chronic obstructive pulmonary disease; SpHb, the noninvasive hemoglobin by pulse CO-oximeter; tHb, laboratory hemoglobin; EtCO_2_, end-tidal carbon dioxide; PaCO_2_, arterial carbon dioxide pressure; PACU, post-anesthesia care unit.

**Table 2:** Accuracy of SpHb by PaCO_2_ range.

	Bias (95% CI) (g/dL)	SD (g/dL)
PaCO_2_ < 35 mmHg	−1.09 (−1.28 to −0.90)	1.06
35 ≤ PaCO_2_ < 45 mmHg	−0.97 (−1.17 to −0.76)	1.22
45 mmHg ≤ PaCO_2_ *	−1.59 (−1.99 to −1.19)	1.22

Bias = the SpHb—laboratory hemoglobin. * *p* = 0.020 compared with PaCO_2_ < 35 mmHg, *p* = 0.004 compared with 35 ≤ PaCO_2_ < 45 mmHg. SpHb, the noninvasive hemoglobin by pulse CO-oximeter; CI, confidence intervals; SD, standard deviation; PaCO_2_, arterial carbon dioxide pressure.

## Data Availability

The datasets used and analyzed during the current study are available from the corresponding author upon reasonable request.
